# The potential of the microbiome as a target for prevention and treatment of carbapenem-resistant Enterobacteriaceae infections

**DOI:** 10.3389/fcimb.2025.1674534

**Published:** 2025-10-08

**Authors:** Lu Zhang, Tinghui Xu, Wenqian Chen, Yinying Chai, Yi Wu, Xinghai Du

**Affiliations:** The First Affiliated Hospital of Zhejiang Chinese Medical University (Zhejiang Provincial Hospital of Chinese Medicine), Hangzhou, Zhejiang, China

**Keywords:** carbapenem-resistant Enterobacteriaceae, gut microbiota, colonization resistance, microbiome-targeted therapy, probiotics, fecal microbiota transplantation, short-chain fatty acids

## Abstract

Carbapenem-resistant *Enterobacteriaceae* (CRE) present an escalating threat to global health due to their high transmissibility, limited treatment options, and high mortality rates. The gastrointestinal tract serves as both a major reservoir and a transmission hub for CRE, especially under conditions of antibiotic-induced dysbiosis. This review highlights the growing interest in the gut microbiome as a potential target for preventing and managing CRE infections. Building upon the understanding of CRE pathogenesis, we examine how commensal microbiota contribute to colonization resistance through mechanisms such as nutrient competition, spatial niche exclusion, immune modulation, and the production of antimicrobial metabolites. We further discuss microbiome-based therapeutic strategies, including probiotic administration, fecal microbiota transplantation (FMT), and supplementation with short-chain fatty acids (SCFAs), that have shown encouraging results in reducing intestinal CRE colonization. In addition, we explore emerging microbiome engineering approaches, particularly CRISPR-Cas9-mediated systems, which enable the selective elimination of resistant strains while maintaining microbial homeostasis. Current microbiome-based approaches have shown promise in the treatment and prevention of CRE infections, but further research is still needed to clarify their mechanisms, evaluate long-term safety, and determine their effectiveness in different clinical settings. With continued studies and thoughtful integration into existing infection control and antibiotic stewardship practices, these strategies may gradually contribute to a more practical and sustainable way to manage CRE.

## 1 Introduction

Carbapenem-resistant *Enterobacteriaceae* (CRE) have become a serious global public health concern. The World Health Organization (WHO) lists carbapenem-resistant *Klebsiella pneumoniae* (*K. pneumoniae*) and *Escherichia coli* (*E. coli*) as critical priority pathogens in its 2024 Bacterial Priority Pathogens List (BPPL), emphasizing their significance in antimicrobial resistance (Sati et al., 2025). These bacteria exhibit a high capacity for acquiring and disseminating resistance genes, making containment and treatment increasingly difficult. CRE cause severe infections such as bloodstream infections, pneumonia, and urinary tract infections. These infections often result in high death rates, longer hospital stays, and higher healthcare costs (Chen et al., 2024; Bhat et al., 2025). Although agents such as polymyxins, tigecycline, and ceftazidime-avibactam are used in the treatment of CRE infections, their roles vary. Polymyxins and tigecycline are often reserved for multidrug-resistant K. pneumoniae, whereas ceftazidime-avibactam is mainly applied to KPC-producing strains. Their clinical utility, however, is increasingly compromised by rising resistance rates, rapid dissemination of resistance genes, and drug-related toxicities (Gao et al., 2025; Hou et al., 2025).

In addition to overt infections, CRE can persist silently in the intestinal tract, particularly in hospitalized and immunocompromised patients (Wu et al., 2023; Xiao et al., 2024; Han et al., 2025). Increasing evidence shows that antibiotic exposure, underlying diseases, and immune suppression can disturb the gut microbiota, lowering colonization resistance and facilitating CRE expansion (Kang et al., 2022; Wang Z. et al., 2024). A disrupted microbial environment not only weakens host defense but also promotes overgrowth of resistant strains. As host immunity declines, CRE may translocate across the intestinal barrier, leading to severe bloodstream infections and other life-threatening complications. The gastrointestinal tract is both a key site of susceptibility and a potential target for new strategies in prevention and treatment. In this context, targeting the gut microbiome offers a promising addition to traditional antibiotics. Methods such as probiotics, fecal microbiota transplantation (FMT), and microbiota-derived metabolites have shown potential in reducing CRE colonization and preventing transmission in both animal models and clinical trials (Gutiérrez-Fernández et al., 2024; Merrick et al., 2025). Key questions remain regarding their mechanisms, long-term effects, and practical use in clinical settings.

This review provides an overview of the challenges posed by CRE in terms of resistance, epidemiology, and colonization, while also evaluating microbiome-based strategies such as probiotics, FMT, and microbial metabolites. The aim is to identify interventions that can complement conventional therapies and inform future clinical management.

## 2 Resistance mechanisms and clinical burden of CRE

CRE are members of the *Enterobacteriaceae* order that exhibit resistance to at least one carbapenem-class antibiotic, such as imipenem, meropenem, or ertapenem (Smith et al., 2025). *K. pneumoniae* and *E. coli* are the most clinically important CRE species. These pathogens are commonly responsible for infections of the bloodstream, respiratory tract, and urinary tract, especially in healthcare-associated settings (Li Y. et al., 2024; Zhong et al., 2025). Carbapenem resistance in *Enterobacteriaceae* is mainly caused by the production of carbapenemases. Among them, KPC, NDM, and OXA-48-like enzymes are the most common. These enzymes are increasingly detected in clinical isolates (Ma et al., 2023; Alvisi et al., 2025). In many cases, the resistance genes are located on plasmids, which promote horizontal gene transfer and often carry virulence factors as well (Heng et al., 2025; Li et al., 2025). In addition to enzyme production, membrane-associated mechanisms also play a critical role. The loss of outer membrane porins, such as OmpK36, limits antibiotic entry. At the same time, efflux systems like the tripartite antimicrobial metabolism system actively pump drugs out of the cell, reducing their effectiveness (Jung et al., 2021; Meekes et al., 2025). These mechanisms frequently act together, leading to broad resistance that extends beyond β-lactams, Aminoglycosides, fluoroquinolones, and even last-line agents like colistin may also be rendered ineffective (Wu et al., 2024; Song et al., 2025).

The global prevalence of CRE continues to rise. Surveillance reports show ongoing transmission of dominant clones, such as ST258 in the Mediterranean and ST11 in Asia, especially within hospital environments (Wang Q. et al., 2024; García-González et al., 2025; Zhang et al., 2025). In intensive care units, colonization rates exceed 20%. Long-term care facilities often struggle with persistent environmental contamination (Wu et al., 2023; Elton et al., 2024). Underdiagnosis is common due to limited surveillance infrastructure and the shortcomings of current screening strategies, allowing ongoing silent transmission (Kedišaletše et al., 2023; Pople et al., 2023). The organism can survive on surfaces and equipment, making infection control difficult. In overcrowded healthcare settings, this persistence helps resistant strains spread more easily (Salomão et al., 2023; Elton et al., 2024). Clinical outcomes of CRE infections remain poor. Mortality often exceeds 40% in bloodstream infections and ventilator-associated pneumonia, especially when appropriate therapy is delayed (Chen et al., 2024; Ruvinsky et al., 2024; Kim et al., 2025). Treatment options are limited and frequently complicated by toxicity. Polymyxins and tigecycline serve as last-resort agents but carry risks of nephrotoxicity and rising resistance, including plasmid-mediated mechanisms (Wu et al., 2024; Xie et al., 2024; Jiang et al., 2025). Newer drugs such as ceftazidime-avibactam and cefiderocol offer broader coverage. Still, treatment failures are common due to rapid emergence of resistance caused by porin mutations and novel carbapenemase variants (Li JW. et al., 2024; Tang et al., 2024; Faxén et al., 2025). The rise of hypervirulent carbapenem-resistant strains further worsens outcomes and limits therapeutic success (Lei et al., 2024; Wang et al., 2025).

Recent efforts in drug discovery have turned to repurposing established antibiotics and testing combination regimens. Fosfomycin has demonstrated synergistic activity with agents such as meropenem, polymyxin B, and colistin, and both experimental and clinical evidence suggest improved outcomes compared with monotherapy (Ribeiro et al., 2023; Katip et al., 2024). Novel therapeutic strategies are also being explored for hypervirulent carbapenem-resistant *K. pneumoniae*. Some isolates carrying both multidrug resistance and hypervirulent traits show unexpectedly attenuated pathogenicity, reflecting the complex relationship between resistance and virulence that may guide future drug development (Ni et al., 2022; Kochan et al., 2023).

## 3 Role of the gut microbiota in CRE colonization

### 3.1 Antibiotic-induced dysbiosis and CRE colonization

Antibiotic exposure is a major risk factor for CRE colonization in the gastrointestinal tract. Short-term, targeted oral antibiotics such as rifampicin can help rapidly decolonize CRE in acute clinical settings, while promoting the enrichment of antagonistic commensals and supporting immune recovery (Ni et al., 2024). However, prolonged or inappropriate use of antibiotics can cause lasting alterations to the gut microbiota, reducing diversity and depleting beneficial bacteria, judicious use of certain agents may help restore a healthier microbial community. Broad-spectrum antibiotics, especially those targeting anaerobic bacteria, disrupt the gut microbiota, reducing commensals and microbial diversity, which weakens colonization resistance (Macareño-Castro et al., 2022). This allows CRE to occupy vacant ecological niches and proliferate. Clinical studies have shown that patients treated with carbapenem, cephalosporin, or fluoroquinolone antibiotics experience significantly higher CRE colonization rates (Sindi et al., 2022; Yuan et al., 2022). Antibiotics also create an environment favorable for CRE growth by depleting microbial metabolites that inhibit its proliferation, while enriching nutrients that CRE can use (Yip et al., 2023). Moreover, dysbiosis promotes the horizontal transfer of resistance genes, turning the gut into a reservoir of multidrug resistance (Rooney et al., 2019; He et al., 2025). The biofilm environment in the gut promotes resistance gene transfer, accelerating the rapid spread of resistance among members of the *Enterobacteriaceae* family, including gene transfer from *E. coli* to *Klebsiella* and from commensals to pathogens (Kent et al., 2020; Michaelis and Grohmann, 2023).

Gastrointestinal colonization plays a central role in the persistence and dissemination of CRE. Long-term shedding of CRE is common in asymptomatic carriers, facilitating its ongoing transmission within hospital wards and intensive care units (Sindi et al., 2022; Baek et al., 2023). The carrier state can persist for up to one year, with approximately 33% of CRE carriers remaining positive after one year (Ciobotaro et al., 2016). Surfaces, devices, and healthcare worker hands often become secondary reservoirs (Deng et al., 2022; Han et al., 2025). Colonization risk is heightened in patients with antibiotic-induced dysbiosis, immunosuppression, or frequent invasive procedures (Khachab et al., 2024; Lee et al., 2024). Repeated antimicrobial exposure further complicates eradication. Standard decolonization approaches are often ineffective, and recolonization occurs frequently. These factors facilitate silent persistence and recurrent infections, even after apparent clearance. Among patients with CRE colonization, approximately 21% developed secondary infections within 180 days of initial colonization, with most occurring within 30 days (Tubb et al., 2025). Consequently, the gut remains a stable reservoir for both endogenous infection and nosocomial transmission (Liu et al., 2022; Sim et al., 2022).

### 3.2 Gut microbiota defense mechanisms against CRE colonization

Healthy gut microbiota confer resistance to colonization by CRE by occupying both nutritional and spatial niches, limiting the resources and ecological space required for pathogen expansion. Commensal bacteria, including species such as Bacteroides, Clostridia and Lactobacillus, engage in exploitative competition by rapidly consuming available monosaccharides, amino acids, and micronutrients, restricting the supply of metabolic substrates necessary for CRE proliferation (Djukovic et al., 2022; Isaac et al., 2022). Meanwhile, mucosa-associated microbial communities form structured biofilms within the inner mucus layer and intestinal crypts, where densely packed bacterial cells and extracellular matrix components create a physical barrier that effectively blocks pathogen access to epithelial adhesion sites (Zhao and Maynard, 2022). These spatially organized structures are stabilized through dynamic interactions between commensal microbes and host-derived mucus, contributing to immune tolerance and sustained exclusion of pathogenic bacteria.

The gut microbiota also plays a crucial role in modulating host immune responses to combat CRE infection. Pattern recognition receptors (PRRs), including NOD1, NOD2, and Toll-like receptors (TLRs), recognize microbial-associated molecular patterns (MAMPs) derived from commensal bacteria, activating downstream signaling pathways that promote the production of antimicrobial peptides (Zhao et al., 2018; Martin-Gallausiaux et al., 2022). In addition, the microbiota regulates cytokine responses by promoting the expression of cytokines such as IL-1β and IL-22, thereby enhancing epithelial barrier function and modulating inflammatory responses (Wu et al., 2022; Zhao et al., 2025).

**Figure 1:**
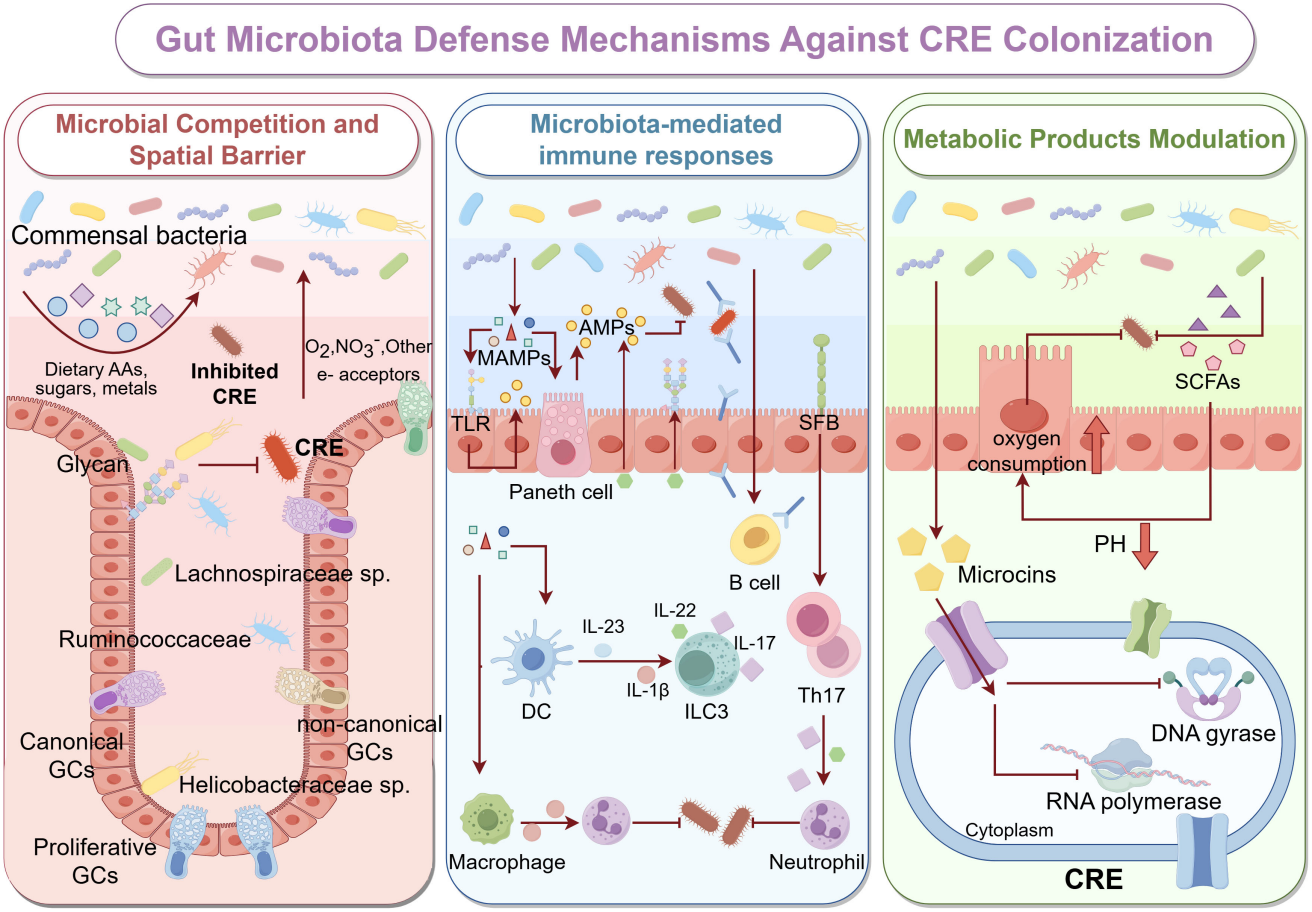
Gut Microbiota Defense Mechanisms Against CRE Colonization The healthy gut microbiota protects against colonization by CRE through three major mechanisms: microbial competition and spatial exclusion (left panel), microbiota-mediated immune responses (middle panel), and metabolic modulation (right panel). left panel: Healthy gut commensals, including *Lachnospiraceae*, *Ruminococcaceae*, and non-canonical GC-associated taxa, establish dense, biofilm-like communities along the intestinal epithelium. These bacteria occupy specific ecological niches and utilize dietary amino acids, complex glycans, and essential micronutrients, thereby reducing the resources available for CRE to proliferate. Spatial organization of microbial aggregates, along with mucin layers secreted by goblet cells, creates physical barriers that limit CRE adherence to epithelial surfaces and hinder their colonization. middle panel: MAMPs, such as lipopolysaccharides, peptidoglycans, and flagellin, are sensed by host PRRs, including TLRs on epithelial and immune cells. This recognition stimulates Paneth cells to secrete AMPs, including defensins and regenerating islet-derived proteins, which directly inhibit CRE growth. Commensal bacteria further modulate innate and adaptive immunity by activating DCs, macrophages, B cells, and type ILC3s, promoting the release of cytokines such as IL-22 and IL-17. These cytokines enhance mucosal barrier integrity, stimulate epithelial proliferation, and recruit neutrophils to sites of potential CRE invasion. right panel: Gut microbiota produce metabolites, such as SCFAs, which lower luminal pH, enhance epithelial oxygen consumption, and create metabolically unfavorable conditions for CRE survival. Additionally, microbiota-derived bacteriocins, including microcins, can penetrate CRE membranes and inhibit key cellular processes, such as DNA gyrase, RNA polymerase activity, and cell wall biosynthesis, leading to targeted suppression.

The metabolic activity of the gut microbiota profoundly shapes the chemical environment of the intestinal lumen, creating conditions that are unfavorable for CRE survival and colonization. Short-chain fatty acids (SCFAs), including acetate, propionate, and butyrate, lower luminal pH and enhance epithelial oxygen consumption, thereby eliminating oxygen-rich niches that favor CRE colonization (Sorbara et al., 2019; Yip et al., 2023). Moreover, butyrate and propionate function as histone deacetylase (HDAC) inhibitors, inducing epigenetic modifications that regulate host gene expression (Korsten et al., 2022). These changes upregulate genes involved in antimicrobial defense, mucin production, and barrier integrity (Pace et al., 2021; Korsten et al., 2023), collectively reducing CRE adhesion to and invasion of intestinal epithelial cells. Studies have shown that certain commensal strains produce narrow-spectrum bacteriocins, particularly microcins, which penetrate the outer membrane of Gram-negative *Enterobacteriaceae* via receptor-mediated uptake, exerting targeted antimicrobial activity and interfering with essential cellular processes such as peptidoglycan synthesis and nucleic acid metabolism (Telhig et al., 2020; Telhig et al., 2022) (**Figure 1**).

## 4 Therapeutic strategies targeting the microbiome against CRE

### 4.1 Probiotic therapy

Probiotic therapy represents a promising microbiome-based strategy against CRE. The antimicrobial effects of probiotics are primarily mediated through the production of acidic metabolites and reprogramming of the gut microbial community. The anti-CRE effect of probiotics was related to the pH-dependent mechanism, and the antibacterial effect was eliminated at pH 7.0 in the upper layer of the cell membrane, but the antibacterial effect remained unchanged after heat treatment (Tang et al., 2023). Lactic acid bacteria produce lactate, organic acids, CO_2_, exopolysaccharides, bacteriocins, and enzymes, which lower the intestinal pH and exert direct antimicrobial effects (Tang et al., 2023; Hatem et al., 2024). Multiple probiotics have been shown to modulate the gut microbiota by exhibiting strong bile salt hydrolase deconjugation and 7α-dehydroxylation activity, leading to increased levels of deoxycholic acid and lithocholic acid, while simultaneously reducing the production of isobutyric acid, isovaleric acid, hydrogen sulfide, and ammonia (Foley et al., 2021; Liu et al., 2024).

Probiotics also enhance host defenses by strengthening the intestinal barrier and modulating mucosal immunity. They promote the gene and protein expression of tight junction proteins Occludin, Claudin, and ZO-1 and stimulate mucin secretion, thereby reinforcing epithelial barrier integrity and reducing pathogen adhesion and invasion (di Vito et al., 2022; Bu et al., 2023). At the same time, probiotics activate the gut mucosal immune system, markedly increasing the levels of secretory IgA, IgA, and IgG in the intestine, while enhancing the functions of CD11c positive dendritic cells and CD4 positive T cells (Lin et al., 2021; Bu et al., 2023). These effects help maintain gut microbial homeostasis and reduce the risk of pathogen translocation and systemic inflammation.

In one screening study of 57 strains, five candidates (LUC0180, LUC0219, LYC0289, LYC0413, and LYC1031) produced inhibition zones larger than 15 mm and sustained suppression of carbapenem-resistant *E. coli* (CRE316) and *K. pneumoniae* (CRE632) (Chen et al., 2019). In addition to these strains, other species including *Bifidobacterium longum* (*B. longum*), *Lactiplantibacillus plantarum* (*L. plantarum*), and *Lacticaseibacillus rhamnosus* (*L. rhamnosus*) have also demonstrated notable antimicrobial activity against CRE, with inhibition zones exceeding 20 mm in representative isolates (Chornchoem et al., 2025). Clinically, a retrospective analysis of ICU patients showed that among 474 individuals receiving probiotics, the incidence of new CRE colonization was significantly reduced, with only 13 patients developing new CRE colonization, compared to a markedly higher rate in the control group (Lee et al., 2023).

### 4.2 FMT

FMT restores colonization resistance against CRE by reestablishing a diverse and balanced gut microbiome following disruption by antibiotics. Antibiotic exposure depletes commensals such as Bifidobacteriaceae and Bacteroidales, exhausts inhibitory metabolites, and enriches the intestinal environment with fermentable nutrients that CRE can exploit for growth (Yip et al., 2023). FMT introduces a complex microbial consortium from healthy donors to restore ecological competition, metabolic inhibition, and spatial exclusion (Millan et al., 2016). Transkingdom interactions between the virome and bacteriome induced by FMT may play a critical role in CRE clearance. Studies have observed a striking increase in *E. coli* phages in carriers of CRE *E. coli* following FMT, as well as concurrent CRE elimination and similar evolutionary patterns of *Klebsiella* phages in mouse models (Liu et al., 2022).

In a study of 10 carriers with prolonged CP-CRE carriage, FMT achieved decolonization rates of 40.0%, 50.0%, and 90.0% within 1, 3, and 5 months after the initial treatment, respectively, especially in patients whose gut microbiota rapidly shift toward the donor composition and have lower baseline *Klebsiella* abundance (Lee et al., 2021). Consistently, another cohort study of 35 patients reported that 68.6% were decolonized within one year after FMT (Shin et al., 2022). Microbiota analyses revealed significant increases in α- and β-diversity metrics in patients with successful CRE decolonization, whereas no such changes were observed in non-responders (Bar-Yoseph et al., 2021). FMT also shows a favorable safety profile. Among the 209 patients reviewed, including immunocompromised individuals, no serious adverse events were attributed to FMT (Macareño-Castro et al., 2022).

### 4.3 Metabolite supplementation therapy

Microbial metabolites, particularly SCFAs, play a key role in the prevention and treatment of CRE. Studies have found that the levels of isobutyric acid and valeric acid are significantly reduced in CRE carriers (Baek et al., 2023). Propionate shows dose-dependent growth inhibition against various multidrug-resistant bacteria, including *E. coli*, with minimum inhibitory concentrations ranging from 10 to 25 mM (Ormsby et al., 2020). Butyrate enhances macrophage antimicrobial activity through HDAC3 inhibition, increasing antimicrobial peptide expression and resistance to enteropathogens (Schulthess et al., 2019). SCFAs also suppress plasmid-mediated resistance gene transfer, with conjugation fully suppressed at concentrations of 0.1–1 M and significant reductions observed even at 0.01 M (Ott and Mellata, 2024). Combining SCFAs with antibiotics yields synergistic effects. SCFAs restore the susceptibility of resistant *Enterobacteriaceae* to β-lactam/β-lactamase inhibitor combinations and downregulate virulence genes including fliC, ipaH, fimH, and bssS (Kadry et al., 2023).

### 4.4 Emerging microbiome engineering technologies

Synthetic biology offers new tools for precisely engineering the gut microbiota to prevent CRE. CRISPR/Cas9 gene editing technology has shown great potential in the prevention and treatment of CRE by targeting carbapenemase genes to reverse resistance. The CRISPR-Cas9-mediated plasmid clearing system has been developed to effectively eliminate carbapenemase genes such as blaKPC, blaNDM, and blaOXA-48, with an efficiency exceeding 94% (Hao et al., 2020). This system has demonstrated excellent results across various clinical isolates of Enterobacteriaceae, including *K. pneumoniae*, *E. coli*, and *Enterobacter cloacae* (*E. cloacae*) (Hao et al., 2020; Tao et al., 2022). This strategy can be used for *in situ* microbiome modification to eradicate targeted resistant and/or pathogenic bacteria without affecting other non-targeted bacterial species. In addition, the delivery of CRISPR-Cas9 by engineered probiotics has achieved over 99.9% elimination of targeted antibiotic-resistant *E. coli* in the mouse gut microbiota with a single dose (Neil et al., 2021). CRISPR-armed phages, which integrate the CRISPR-Cas system, enable precise targeting and killing of *E. coli*, targeting bacteria in biofilms and reducing the emergence of antibiotic-resistant strains (Gencay et al., 2024) (**Table 1**).

**Table 1:** Therapeutic Strategies Targeting the Microbiome Against CRE.

Therapeutic strategy	Specific therapy	Mechanism	Clinical effect or research progress
Probiotic Therapy	Lactobacilli (LUC0180, LUC0219, LYC0289, LYC0413, and LYC1031)	The production of organic acids by probiotic organisms and the resulting decrease in culture pH	Lactobacillus strains at a concentration of 108 CFU/ml totally inhibited the growth of CRE316 and CRE632 after a 24-h incubation.
	*B. longum*, *L. plantarum*, and *L. rhamnosus*	Reduce the secretion of pro-inflammatory cytokines while enhancing the production of anti-inflammatory cytokines	Exhibited strong antimicrobial activity, with inhibition zones greater than 20 mm against antibiotic-resistant strains, including CRE
	Saccharomyces boulardii and Lactobacillus rhamnosus	Produce antimicrobial compounds, occupy epithelial niches, and limit pathogen colonization	Among 474 patients receiving probiotics, only 13 developed new CRE colonization, significantly fewer than in the control group.
FMT	Administration via a colonoscopy or esophagogastroduodenoscopy	FMT altered microbiota composition, increasing Bacteroidetes and reducing CRE-related genera	90% of CRE carriers achieved decolonization within 5 months of FMT, with 40% clearing CRE within the first month
	Administration via a gastro-endoscope or colonoscopy	FMT promotes CRE decolonization by restoring gut microbiota balance and reversing dysbiosis	In a cohort of 35 patients, 68.6% achieved decolonization within one year, with FMT and multidrug-resistant organisms type identified as key factors influencing decolonization time.
	Oral capsulized FMT	Restore the gut microbiome and compete with residual resistant strains	Participants who achieved CRE eradication, reaching 66.7% at 6 months, exhibited significant shifts in both α- and β-diversity metrics, changes not observed in non-responders.
Metabolite Therapy	Butyrate	Exerted HDAC3 inhibition to alter metabolism and induce antimicrobial peptide production	Exhibit strong antimicrobial activity, thereby reducing the spread of pathogenic bacteria.
	SCFAs	Inhibit bacterial plasmid transfer	Reductions in transconjugant populations were observed in all three SCFA groups, effectively eliminating antimicrobial resistance with minimal impact on bacteria.
	SCFAs and β-lactam/β-lactamase inhibitor combination synergy	Restore susceptibility to β-lactam/β-lactamase inhibitor and suppress virulence genes	The addition of SCFAs increased the susceptibility of the 18 tested isolates, achieving rates of 94.4%, 83.3%, and 66.7% for ceftazidime/avibactam, cefoperazone/sulbactam, and cefepime/enmetazobactam, respectively.
Synthetic Biology	CRISPR-Cas9-mediated plasmid-curing system	Eliminate carbapenemase genes and plasmids in clinical *Enterobacteriaceae* isolates	Effectively cured clinical isolates from various *Enterobacteriaceae* species with a clearing efficiency of over 94%, resensitizing CRE to carbapenem antibiotics.
	Engineered probiotics with antibacterial CRISPR-Cas	Eliminate a target strain from a mixed population and protect the microbiota from specific strain colonization	The conjugative delivery system eliminates over 99.9% antibiotic-resistant *E. coli* in the mouse gut with a single dose.
	Engineered phage with antibacterial CRISPR-Cas	Precisely lyse target bacteria and disrupt resistance genes to enhance strain-specific clearance	Enable precise targeting and killing of *E. coli*, including bacteria in biofilms, while reducing antibiotic resistance emergence.

CRE, Carbapenem-Resistant Enterobacteriaceae; FMT, Fecal Microbiota Transplantation; SCFA, Short-Chain Fatty Acid; HDAC3, Histone Deacetylase 3; CRISPR, Clustered Regularly Interspaced Short Palindromic Repeats; Cas, CRISPR-associated; B. longum, *Bifidobacterium longum*; *E. coli, Escherichia coli; L. plantarum, Lactiplantibacillus plantarum; L. rhamnosus, Lacticaseibacillus rhamnosus*.

## 5 Conclusion

The global rise of CRE presents a critical challenge to infection control, driven by complex resistance mechanisms, asymptomatic gastrointestinal colonization, and limited treatment options. The gut serves as both a reservoir and a transmission hub for CRE, particularly under conditions of antibiotic-induced dysbiosis that impair colonization resistance and facilitate horizontal gene transfer. In this context, the gut microbiota has emerged as a promising therapeutic target.

Microbiome-based interventions such as probiotics, FMT, and SCFA supplementation have shown potential to restore microbial balance and suppress CRE colonization. Most current evidence, however, is derived from *in vitro* experiments and animal models, with only limited support from small-scale or retrospective clinical studies. These findings suggest potential preventive and therapeutic value, but their clinical efficacy and safety remain to be rigorously validated in large, well-designed randomized controlled trials. While traditional approaches may provide broad-spectrum benefits, precision tools such as CRISPR/Cas9 gene editing and engineered probiotics represent a highly innovative frontier. These technologies hold the promise of selectively removing resistance genes while minimizing collateral disruption to commensal microbes. Yet, their translation into clinical practice is still at the proof-of-concept stage, with substantial barriers including the development of reliable delivery systems, managing potential off-target effects, and navigating complex regulatory pathways for live biotherapeutics.

Moreover, inter-individual variability in baseline microbiota may influence treatment outcomes, underscoring the importance of personalized approaches. For these advanced strategies to succeed, they must ultimately be integrated into established infection control frameworks, including patient screening, contact precautions, environmental hygiene, and antimicrobial stewardship. A coordinated and evidence-based strategy that bridges microbiome-targeted therapies with existing infection control practices will be essential to move from reactive treatment toward proactive and sustainable CRE containment.
